# Long-Range α-Synchronization as Control Signal for BCI: A Feasibility Study

**DOI:** 10.1523/ENEURO.0203-22.2023

**Published:** 2023-03-02

**Authors:** Martín Esparza-Iaizzo, Irene Vigué-Guix, Manuela Ruzzoli, Mireia Torralba-Cuello, Salvador Soto-Faraco

**Affiliations:** 1 Center for Brain and Cognition, Universitat Pompeu Fabra, Barcelona, Spain 08005; 2 Basque Center on Cognition Brain and Language (BCBL), Donostia–San Sebastián, Spain 20009; 3 Ikerbasque, Basque Foundation for Science, Bilbao, Spain 20009; 4 Institució Catalana de Recerca i Estudis Avançats (ICREA), Barcelona, Spain 20009

**Keywords:** Alpha, brain-computer interface, EEG, oscillations, phase coupling, visuospatial attention

## Abstract

Shifts in spatial attention are associated with variations in α band (α, 8–14 Hz) activity, specifically in interhemispheric imbalance. The underlying mechanism is attributed to local α-synchronization, which regulates local inhibition of neural excitability, and frontoparietal synchronization reflecting long-range communication. The direction-specific nature of this neural correlate brings forward its potential as a control signal in brain-computer interfaces (BCIs). In the present study, we explored whether long-range α-synchronization presents lateralized patterns dependent on voluntary attention orienting and whether these neural patterns can be picked up at a single-trial level to provide a control signal for active BCI. We collected electroencephalography (EEG) data from a cohort of healthy adults (*n* = 10) while performing a covert visuospatial attention (CVSA) task. The data show a lateralized pattern of α-band phase coupling between frontal and parieto-occipital regions after target presentation, replicating previous findings. This pattern, however, was not evident during the cue-to-target orienting interval, the ideal time window for BCI. Furthermore, decoding the direction of attention trial-by-trial from cue-locked synchronization with support vector machines (SVMs) was at chance level. The present findings suggest EEG may not be capable of detecting long-range α-synchronization in attentional orienting on a single-trial basis and, thus, highlight the limitations of this metric as a reliable signal for BCI control.

## Significance Statement

Cognitive neuroscience advances should ideally have a real-world impact, with an obvious avenue for transference being brain-computer interface (BCI) applications. The hope is to faithfully translate user-generated brain endogenous states into control signals to actuate devices. A paramount challenge for transfer is to move from group-level, multitrial average approaches to single-trial level. Here, we evaluated the feasibility of single-trial estimation of phase synchrony across distant brain regions. Although many studies link attention to long-range synchrony modulation, this metric has never been used to control BCI. We present a first attempt of a synchrony-based BCI that, albeit unsuccessful, should help break new ground to map endogenous attention shifts to real-time control of brain-computer actuated systems.

## Introduction

A few decades ago, imagining an interface between the human brain and a computer was closer to science fiction than to scientific achievement. Nowadays, brain-computer interfaces (BCIs) can read out brain activity, extract features from the signal in real-time, and convert them into outputs for monitoring, controlling devices, or even modifying cognitive states (Blankertz et al., 2016). One significant challenge of BCIs is finding reliable control signals from brain activity with a sufficiently high signal-to-noise ratio (SNR) at a trial-by-trial level to allow successful classification. Ideally, the Ocurrence of the target brain activity should depend on endogenous mental states that a user can control at will. The use of noninvasive, cost-effective, and light-weight neuroimaging devices can, in turn, facilitate transfer to real-life applications. For now, electroencephalography (EEG) is the most viable candidate to achieve real-life BCI.

For example, some EEG-based BCIs have used motor imagery as a control signal (e.g., imagined right/left-limb movement; Padfield et al., 2019), whereas others have used neural correlates of covert visuospatial attention (CVSA; van Gerven and Jensen, 2009; Treder et al., 2011; Tonin et al., 2013). Here, we will concentrate on the latter. In human behavior, CVSA is used to direct processing resources to relevant locations in the environment while disengaging from irrelevant locations (Pashler, 1999; Foster and Awh, 2019). CVSA can be manipulated through a Posner cueing protocol (Posner, 1980), which shows a robust effect on behavioral performance: higher accuracy and faster reaction times for targets appearing at the cued (attended) location compared with targets appearing in un-cued, putatively unattended locations (Posner, 1980).

Shifts in CVSA are associated with changes in oscillatory activity in the α band (α, 8–14 Hz) at parieto-occipital regions (Klimesch, 1999; Foster et al., 2017). Typically, α-power shows an interhemispheric imbalance when attention is covertly oriented to either the left or right visual field, revealing its potential as a control signal for BCI implementations (Thut et al., 2006; Rihs et al., 2007; for a review, see Astrand et al., 2014b). Interhemispheric α-power imbalance corresponds to a late process in CSVA shifts (van Diepen et al., 2019). First, cueing information is integrated through sensory pathways in a bottom-up fashion, reaching higher visual areas in the parietal cortex [e.g., intraparietal sulcus (IPS)] and eventually frontal regions [e.g., frontal eye fields (FEFs); Petersen and Posner, 2012]. From there on, top-down modulation shifts attention to the corresponding hemifield, where it is maintained during target anticipation (Simpson et al., 2011). The mechanism involved in this top-down modulation is thought to involve long-range α-synchronization between the frontal and posterior cortex, which eventually leads to classical interhemispheric imbalances in α-power observed in the visual cortex (Sauseng et al., 2005; Doesburg et al., 2009; Lobier et al., 2018). Long-range synchronization is a potential mechanism to increase the fidelity and effectiveness of communication throughout the brain (Clayton et al., 2018) among occipital, parietal, and frontal regions (Sadaghiani and Kleinschmidt, 2016). Synchronizing excitability cycles between distant neural populations increases the likelihood of spikes from one region discharging postsynaptic potentials during a specific (excitable) phase of the other (Fries, 2015). Despite the evidence supporting this model (Buschman and Miller, 2007; Cardin et al., 2009), there is still debate on its temporal dynamics, lateralization patterns and individual-level variability.

Despite the evidence of links between long-range α-synchronization and behavioral performance in group-level analyses (Sauseng et al., 2005; Doesburg et al., 2009, 2016), BCI protocols based on endogenous attention orienting have only used α-power as a control signal. In our study, we attempt to replicate a previously demonstrated effect in attention orienting involving long-range α-synchronization to assess its feasibility in BCI paradigms. The original publication (Sauseng et al., 2005) found significant increases in contralateral over ipsilateral connectivity around the time of target appearance. We hypothesized that, if attention-driven connectivity emerged in target-centered time windows, it may also be present in the cue-to-target interval, where participants are putatively shifting attention toward the cued side. Further, this cue-to-target time window would enable the use of long-range α-synchronization in BCIs based on purely endogenous brain signals. Therefore, we will test whether such contralateral and ipsilateral patterns in α-synchronization emerge in single-trial dynamics with sufficient signal strength to make them a reliable control signal. To do so, we used an EEG dataset from a lateralized endogenous spatial attention task to replicate the group-level effects found by Sauseng et al. (2005), to explore the cue-to-target interval, and to classify the direction of attention at the single-trial level using long-range α-phase synchronization as proof of concept for transference to BCI.

## Materials and Methods

### Participants

We used data from a previous, unrelated study (Torralba et al., 2016). The dataset consisted of 15 participants (mean age = 22, SD = 3; 7 female). All participants provided informed consent and had a normal or corrected-to-normal vision. The study was run in accordance with the Declaration of Helsinki and the experimental protocol approved by the local ethics committee CEIC Parc de Salut Mar (Barcelona, Spain).

### Task

**Figure 1.**
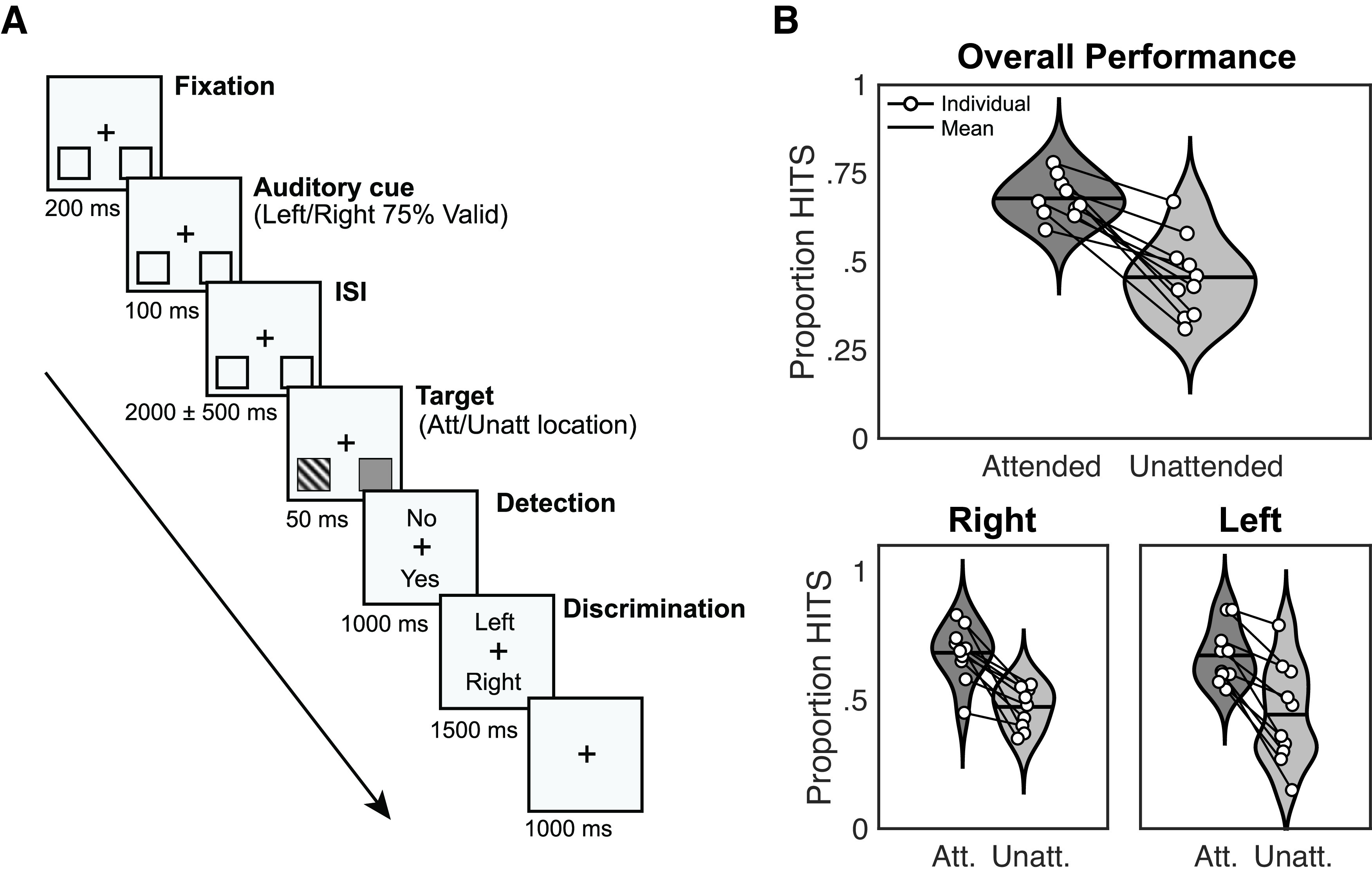
Experimental design and response rates. ***A***, Schematic trial representation. A black fixation cross in the middle of the screen and two squares (to-be-attended locations) at the bottom left, and bottom right positions were displayed continuously. At the beginning of each trial, participants were instructed to gaze at the fixation cross. After 200 ms (fixation period), an auditory cue appeared for 100 ms (cue period) indicating which hemifields participants must attend (75% validity). After a jittered interstimulus interval of 2000 ± 500 ms, a target appeared at the targeted location during 50 ms (target period). Participants had to report first if they had seen the target (detection task), and after 1000 ms, the location of the target (left/right discrimination task) during 1500 ms. An intertrial interval (ITI) of 1000 ms followed, and a new trial began (adapted from Torralba et al., 2016). ***B***, Response rates for detected and discriminated trials (HITS) related to attended and unattended trials. Black lines over violin plots represent the mean value. Both overall performance (top) and right/left hemifields (bottom) are shown. White dots indicate individual values (adapted from Torralba et al., 2016).

Before the experimental session, the participant’s EEG activity was recorded during a 5-min recording at rest with eyes closed to extract the individual α frequency (IAF) used in the analyses. In the experimental session, participants performed a modified version of the Posner cueing task (see Fig. 1*A*). The trial started with the onset of a central fixation cross, placed between two placeholders located 20° of visual angle left and right off-center, vertically shifted 20° of visual angle below the fixation cross (see Fig. 1*A*). After a 200-ms fixation period, a central auditory cue (100-ms duration) indicated the likely target location through either high pitch (2000 Hz) or low pitch (500 Hz) tones, the mapping was randomized across subjects. Participants should covertly attend to the indicated side, without moving their eyes, during a jittered interstimulus interval (ISI; 2000 ± 500 ms). The use of a jittered ISI was employed to avoid participants using automatic temporal attention to solve the task. Next, the target (a Gabor grating tilted 45° left or right, 50-ms duration) appeared briefly inside one of the placeholders, with 75% validity regarding the cued location. The grating contrast was adjusted individually, as described below. A noise pattern with an equal overall luminance as the target was presented at the alternative placeholder, with the exact timings as the target. Participants were asked first to indicate if they had detected the target (yes/no detection) and, subsequently, the target’s tilt (left/right discrimination). Both answers were made by keypress, in an un-speeded fashion, and with response mapping (top-bottom) orthogonal to the attention manipulation and varied from trial to trial. A trial was considered correctly answered only when participants both detected the stimulus and discriminated the hemifield in which it was presented. An intertrial interval of 1000 ms followed the response, and a new trial began. Unless otherwise noted, the EEG analyses were done on validly cued trials that were responded correctly. On average, 289.9 ± 11.3 trials from each participant were employed for the EEG analysis.

The Gabor gratings used as stimuli were 0.002 cycles per degree, with a size of 3.35°, embedded in white noise. The contrast was adjusted individually using a preliminary threshold titration procedure in which the Gabor contrast thresholds for both hemifields (left and right) were independently adjusted to a 70% detection rate when cued (i.e., in the attended location). Stimuli were presented on a 21-inch CRT screen with a refresh rate of 100 Hz and a resolution of 1024 × 768 pixels. The experiment was implemented in MATLAB R2015b (MATLAB, RRID: SCR_001622) using the Psychophysics Toolbox (Psychophysics Toolbox, RRID: SCR_002881).

### EEG recording and preprocessing

EEG recordings were obtained from 64 Ag/AgCl electrodes positioned according to the 10–10 system with AFz as ground and nose tip as reference. Impedance was kept below 10 kΩ. The employed system was an active actiCHAmp EEG amplifier from Brain Products. The signal was sampled at 500 Hz and processed in MATLAB 2020 and 2015 (MATLAB, RRID: SCR_001622) using custom functions and the FieldTrip toolbox (FieldTrip, RRID: SCR_004849).

Manual artifact rejection was applied to discard trials where any EOG components had an amplitude higher than 50 μV. Defective channels were repaired using neighbours calculated by triangulation and splines for interpolating channel data. Then, the data were demeaned and notch filtered at 50 Hz to exclude line noise. Next, fifth-order high-pass and sixteenth-order low-pass IIR Butterworth filters were employed to limit the signal between 0.16 and 45 Hz (Sauseng et al., 2005). The filtering was done forward and backwards (two-pass), which resulted in zero phase lag.

### Time-frequency analysis

We performed long-range synchronization analyses in two time windows. The first was time-locked to the target onset (target-locked) to replicate Sauseng et al. (2005) methods and validate our analysis pipeline. The second was time-locked to the cue onset (cue-locked) to estimate long-range α-phase synchronization during covert visuospatial attention shifts.

Following Sauseng et al. (2005), for the target-locked analysis, we used two windows of 200 ms: a pretarget (−200–0 ms) and a post-target window (200–400 ms). The latter excludes the interval 0–200 ms, most affected by the phase resetting effect of target presentation. For the cue-locked analysis, we used the cue-to-target time window between 500 and 1500 ms postcue and divided it into five consecutive and nonoverlapping 200-ms windows. By analysing from 500 ms onwards we avoid the event-related potential (ERP) caused by cue presentation and allow endogenous attention shift to build up, a process which takes a few hundreds of milliseconds (Foxe and Snyder, 2011). The cue-locked analysis period ends at 1500 ms, which was the minimum possible duration of the cue-to-target interval (duration of 2000 ± 500 ms; see Materials and Methods). All epoched data were mirror-reflected to avoid edge artefacts (Cohen, 2014) when performing the time-frequency analysis. Afterwards, data were trimmed, and reflected edges were removed.

We computed the Fourier coefficients using five-cycle Morlet wavelets (Grossmann and Morlet, 1984) with 16 logarithmically spaced frequencies ranging from 2.6 to 42 Hz. For the analysis aimed at replicating Sauseng’s results, we only used wavelets within the upper α-band (9.54–14.31 Hz; Sauseng et al., 2005), whereas, for the exploratory analysis, we used the whole frequency range (i.e., 2.6–42 Hz) to explore further long-range α-phase synchronization in other frequency bands beyond the IAF.

### Connectivity measures

Three clusters of electrodes of interest (EOI) were defined for the connectivity analyses, mimicking Sauseng et al. (2005): A fronto-medial (FM) EOI cluster (Fz, FC1, FC2) and two symmetric posterior clusters located either at the parietal left (PL) region (P3, PO3, PO1) or the parietal right (PR) region (P4, PO4, PO2). To infer connectivity between each parietal EOI cluster and the FM location, we used phase locking value (PLV; Lachaux et al., 1999). This metric estimates the consistency of phase differences between two locations across multiple trials and is not affected by power differences. Mathematically, the PLV is expressed as the absolute value of the average complex unit-length phase differences:

(1) $$PLV(x,y)=\left|\frac{1}{n}\overset{n}{\underset{k=1}{\sum }}e^{i\left(\varphi_{x}\left(k\right)-\varphi_{y}(k)\right)}\right|,$$

where *n* corresponds to the total number of trials indexed by *k* and $\varphi_{x}$, $\varphi_{y}$ correspond to the phases at electrodes *x* and *y,* respectively. PLV was calculated according to Equation 1 using the phases for every combination of individual electrode pairs of the FM-PR and FM-PL networks. Then, these values were averaged, resulting in a time series of PLV FM-PR and FM-PL networks for each of the frequencies of interest and condition (attended left and attended right) trials. Subsequently, the PLV time series were collapsed as either ipsilateral (FM-PL network and attend left; FM-PR and attend right) or contralateral (FM-PR network and attend left; FM-PL and attend right). Therefore, for each participant and frequency of interest, two time series of PLV were obtained (contralateral and ipsilateral PLV).

### Classification

The trial classification was performed using support vector machines (SVMs). We selected FM-PR and FM-PL connectivity as input to the SVM. Attended right and attended left labels for each trial were provided as ground truth for the algorithm. The main goal of the classifier was to infer, on each trial, whether a participant was attending to the left or right hemifield, based on the long-range α-phase synchronization in the left and right frontoparietal networks. Note that PLV is computed across trials, and SVM aims to classify on a single-trial basis, so PLV was also calculated across time points (Cohen, 2015). As a validation step, we repeated the target-locked analysis employing this metric (i.e., cross-time PLV) before proceeding with the cue-locked classification attempt.

We divided the cue-locked interval ranging from 500 to 1500 ms in bins of 200 ms, yielding five values for FM-PR connectivity and five for FM-PL connectivity. The resulting ten values were used as input to the SVM to perform the optimization and classification of the trials. Note that for the classification, we used the data from the participant that achieved a significant difference in PLV values between parietal left and right EOI clusters in all cue-to-target windows (Participant (P)10). Trials were split into a training (80%) and testing (20%) set of trials to avoid overfitting. Then, the training set was subdivided into sub-training (80%) and validation sets (20%).

Our initial approach was to use a linear kernel for the classification. However, after evaluating the option through cross-validation of the validation set and obtaining a negative result (i.e., classification was not better than chance), we decided to use a Gaussian kernel (i.e., Radial Basis Function). In order to select the most suitable and efficient values for classifying attended left and attended right trials from the validation set, we optimized the parametric space of the SVM. This comprised margin and γ parameters, which were explored in logarithmic steps from 10^−6^ to 10^3^ for both constants and every fold.

### Interhemispheric power imbalance analysis

Besides calculating the long-range α-phase coupling, we also computed the interhemispheric α-power imbalance at parietal regions, both at the individual and at group-level, as a reality check. For this reality check, we used Thut et al. (2006) for guidance to choose the electrodes of interest. First, we performed an independent component analysis (ICA, Makeig et al., 1995), during which 3 ± 1 components were discarded on average per participant, based on a visual inspection, the components’ topography, and time course. The rejected components comprised both ocular and motor artifacts. Please note that ICA was only performed for the power analysis, not for the connectivity pipeline, to replicate the exact preprocessing as seen previously (Sauseng et al., 2005) and, importantly, because phase of electrophysiological recordings is affected when ICA components are rejected (Thatcher et al., 2020).

The frequency of interest used in lateralization analyses was adjusted for each participant depending on the individual α frequency (IAF) extracted from the 5-min recording (eyes closed) previous to the experiment (see above). The IAF was determined based on the presence of a single peak (i.e., a local maximum) within the considered frequency band of interest (5–15 Hz) on the power spectrum density (PSD). A spectrogram was extracted for each parieto-occipital electrode (P7, P5, P3, P1, Pz, P2, P4, P6, P8, PO3, PO4, POz, PO9, PO10, O1, Oz, O2) using the Welch method (segments of 1000 ms with a 10% overlap, a Hanning taper to avoid spectral leakage and 0.25-Hz frequency resolution). The power spectrum was averaged across electrodes for each participant and normalized by the mean power from 1 to 40 Hz (Vigué‐Guix et al., 2022).

To extract the α-power during the task, we selected the epoch from −1.5 to 3 s in cue-locked trials by convolving the EEG signal with a set of complex Morlet wavelets (Grossmann and Morlet, 1984) of five cycles (n_c_). The frequencies of the wavelets ranged from IAF ± 1 Hz, in 1-Hz steps. For instance, an IAF peak of 10 Hz would have a bandwidth ranging from 8.33 to 11.67 Hz. Power was extracted from two symmetric regions of interest precisely in PR (P6, P8, PO4, O2) and PL locations (P5, P7, PO3, O1) to replicate as closely as possible the original EOI electrodes used previously (Thut et al., 2006). Power imbalance was computed according to the formula:

(2) $$Lateralization\;Index=\frac{\alpha \left(PR\;EOI\right)-\alpha (PL\;EOI)}{meanof\alpha (PL\;EOI+PR\;EOI)},$$

where α (PL EOI) and α (PR EOI) are the average of α-power over left and right electrodes of interest, respectively. Equation 2 leads to smaller (negative) values where α-activity is more prominent over the left hemisphere than the right [α (PL EOI) > α (PR EOI)] and to larger (positive) values for the opposite pattern [α (PL EOI) < α (PR EOI)]. According to theory and previous findings, values of LI reflecting attention directed to the right hemifield should be larger than LI values reflecting leftward directed attention.

Finally, we also checked whether there was any relationship between the α-power imbalance and the contra-ipsi difference of PLV for each attended location. We explored the correlations between α-lateralization indexes and the effect in PLV contra-ipsi differences at the pretarget (−200–0 ms) and post-target (200–400 ms) windows using Pearson correlations.

### Statistical analyses

A one-tailed nonparametric Monte Carlo permutation test was computed to determine significant differences in PLV between networks for each attended location (Mostame et al., 2019). For each participant, the attended right or left labels were randomly assigned to trials, and surrogate PLVs were calculated from the resulting dataset. This process was repeated 10 000 times (iterations) to create a null distribution of PLV values. The obtained *p*-value corresponded to the proportion of surrogate iterations with a contra-ipsi difference larger than the actual measured value (one-tailed test). This process was performed on every time window defined in the previous section. For the group analysis, the procedure was equivalent, but surrogate PLV distributions were averaged across participants before the statistical test.

For the statistical assessment of the α-power imbalance over time between attended left and attended right trials, we performed a cluster-based permutation test procedure (100,000 randomizations) for each participant and at the group-level (one-tailed permutation test; Maris and Oostenveld, 2007; Meyer et al., 2021). We assessed that lateralization indexes for attended right and attended left trials were two significantly different distributions by applying a one-tailed *t* test (independent samples) with α-level = 0.05 for each participant. At group-level, we performed a one-tailed paired *t* test with the mean lateralization indexes for attended right and attended left trials for each participant with α-level = 0.05. Correlations between α-power imbalance and the contra-ipsi difference of PLV were corrected for multiple comparisons by applying the false discovery rate (FDR) of Benjamini and Hochberg (Benjamini and Hochberg, 1995).

## Results

### Behavioral results

Five participants who presented equivalent detection and discrimination rates for stimuli appearing at cued and un-cued locations were discarded from the analysis, leaving a total of 10 participants. As expected, behavioral results showed that the detection rate calculated based on both the detection response (yes/no) and the discrimination response (left/right; chance level at 0.25) was superior for cued (attended) trials 0.68 ± SEM = 0.02 compared with un-cued (unattended) ones 0.46 ± 0.04 (see Fig. 1*B*). The pattern on each hemifield was equivalent: on the left hemifield attended = 0.68 ± 0.03 and unattended = 0.47 ± 0.03; for the right hemifield attended = 0.67 ± 0.03, and unattended = 0.44 ± 0.06. We used one tailed *t* tests to assess that performance was above chance level (25%) for each of the conditions (attended and unattended) and hemifields separately: attended left trials (0.68 ± 0.11, *p*-value = 2 × 10^−7^, *t*_(9)_ = 12.593), attended right trials (0.67 ± 0.11, *p*-value = 3 × 10^−7^, *t*_(9)_ = 12.226), unattended left trials (0.47 ± 0.08, *p*-value = 5 × 10^−6^, *t*_(9)_ = 8.876) and unattended right trials (0.44 ± 0.20, *p*-value = 0.06, *t*_(9)_ = 3.117).

### Target-locked long-range α-synchrony

**Figure 2.**
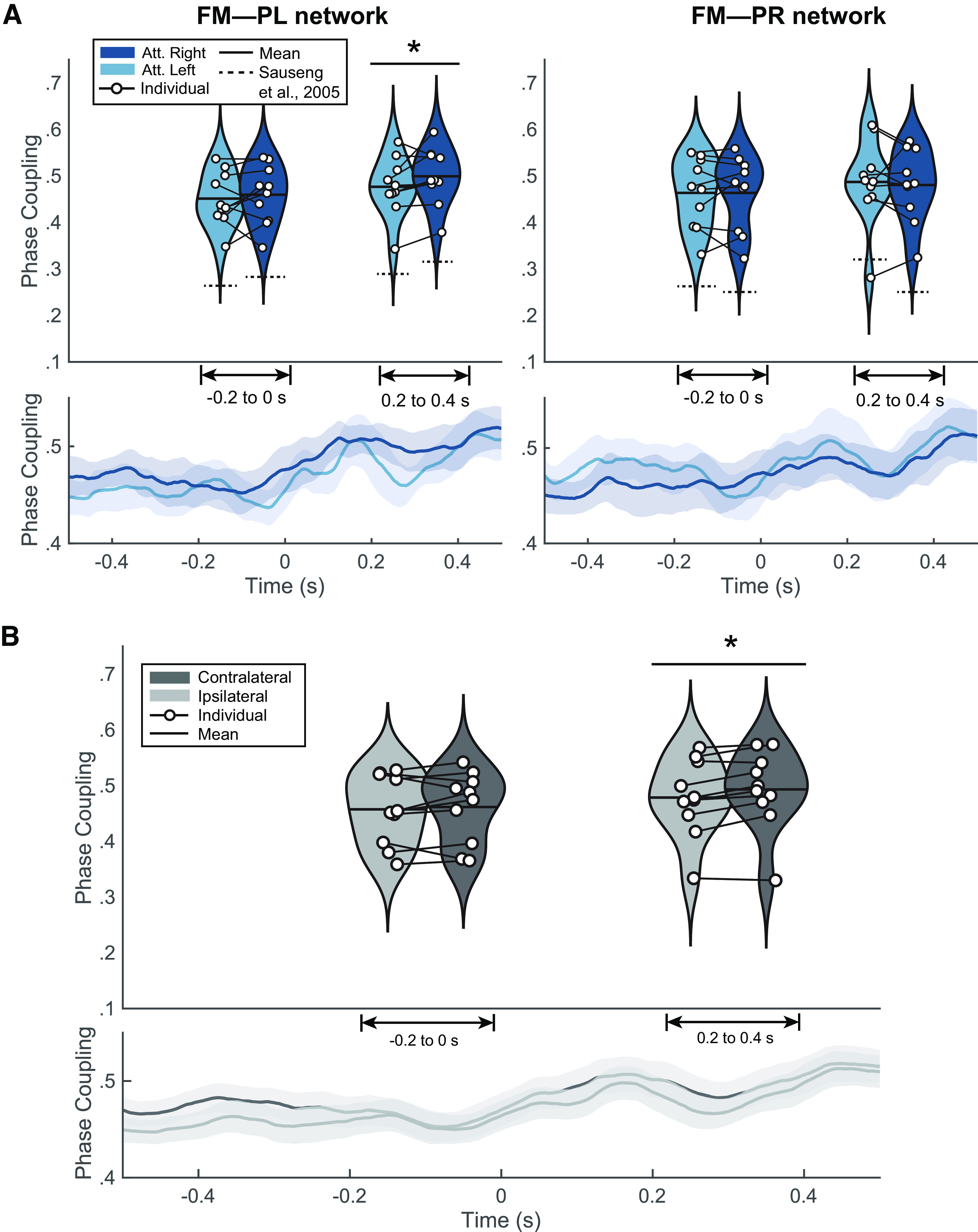
Target-locked results. ***A***, Target-locked results of the phase-coupling for attended left (light blue) and attended right (dark blue) in fronto-medial to parietal left (FM-PL) and fronto-medial to parietal right (FM-PR) networks. The lower panels depict the cross-trial average time course (± shaded standard error of the mean; SEM) of phase-locking value (PLV) in both conditions (attended left and attended right). Upper panels present the binned violin plots (mean and median) of the pretarget window (−200–0 ms) and the post-target window (200–400 ms); *p <* *0.05. ***B***, Target-locked results collapsed as either ipsilateral (FM-PL network and attended left; FM-PR and attended right) or contralateral (FM-PR network and attended left; FM-PL and attended right). The lower panel shows the cross-trial average time course (± shaded SEM) of PLV in ipsilateral (light gray) and contralateral (dark gray) conditions. The upper panel exhibits the distribution of individual PLV with a violin plot, superimposed by the mean and the contralateral to ipsilateral differences between individual PLV; *p <* *0.05. Individual results with PLV are found in Extended Data Figure 2-1, and those with phase linearity measurement (PLM) are found in Extended Data Figure 2-2.

Here, we describe the results from the target-locked analysis, conducted to reproduce Sauseng et al. (2005)’s findings. Long-range synchrony was estimated using PLV between frontal EOI and each of two lateralized parietal EOI. Figure 2 shows the group-level connectivity analysis of the upper α-band (9.54–14.31 Hz). Phase coupling is depicted as the mean across the pretarget window (−200–0 s) and the post-target window (200–400 ms), as well the time course (from to −500 to 500 ms). Regarding the left frontoparietal network (Fig. 2*A*, left), PLV was consistently higher when attention was directed rightward (contralateral) than leftward (ipsilateral) in both pretarget and post-target windows, although the PLV difference only reached significance in the post-target window (*p *<* *0.05). Regarding the right network (Fig. 2*A*, right), PLV was stronger when attention was directed leftward (contralateral) than rightward (ipsilateral) in the post-target window, whereas the pretarget window does not show this difference. Neither window, however, emerged as significant. This pattern generally replicates Sauseng et al. (2005) results, as indicated by the dashed lines in Figure 2*A* representing the mean phase-coupling from their study. Lower panels in Figure 2*A* display the temporal course of phase coupling to provide a time-resolved illustration of the phase-coupling effect. For the attended right condition, PLV values in the left network should be higher than PLV values for the attended left. The inverse pattern should hold in the right network. Moreover, Figure 2*B* presents the PLV with side of attention collapsed as contralateral and ipsilateral with respect to the corresponding network. Individual PLV values, marked as black dotted lines, exhibit a consistent contralateral to ipsilateral increase in the post-target window. Group-level statistical analysis further showcased a significant difference limited to this time window (200–400 ms, *p < *0.05). This result was controlled by avoiding the preprocessing bandpass filter which may affect phase estimation, and by computing a Hjorth filter to avoid the effects of volume conduction (Hjorth, 1975). Both analyses maintained the significant differences between contralateral and ipsilateral PLV (*p *<* *0.05).

10.1523/ENEURO.0203-22.2023.f2-1 Extended Data Figure 2-1 Individual results of target-locked PLV index. Violin plots represent the phase locking values (PLVs) averaged over the pretarget (−200–0 ms, *t* = 0 as target appearance) and post-target time window (200–400 ms). Ipsilateral (FM-PL network and attended left; FM-PR and attended right) or contralateral (FM-PR network and attended left; FM-PL and attended right) scenarios are exhibited as either light grey or dark grey, respectively. *p <* *0.05, **p <* *0.01, ***p <* *0.001. Download Figure 2-1, EPS file.

10.1523/ENEURO.0203-22.2023.f2-2 Extended Data Figure 2-2 Individual results of target-locked PLM index. Violin plots represent the phase linearity measurement (PLM) over the pretarget (−200–0 ms, *t* = 0 as target appearance) and post-target time window (200–400 ms). Ipsilateral (FM-PL network and attended left; FM-PR and attended right) or contralateral (FM-PR network and attended left; FM-PL and attended right) scenarios are exhibited as either light grey or dark grey, respectively. *p <* *0.05, **p <* *0.01, ***p <* *0.001. Download Figure 2-2, EPS file.

At individual level, only three out of 10 participants showed significant contralateral PLV increase (P02, *p < *0.01; P05, *p < *0.01; P07, *p < *0.01; see Extended Data Fig. 2-1). The lack of a significant group-level effects in the pretarget window is consistent with individual phase coupling, as a multiple subject present a trend in the opposite direction as expected (i.e., ipsilateral over contralateral PLV; see Extended Data Fig. 2-1). We further assessed single-subject synchronization through the phase linearity measurement (PLM) as it has been recently reported to be a robust metric for trial-level connectivity (Baselice et al., 2019). We did not find any significant effects in any participant (*p *>* *0.05; see Extended Data Fig. 2-2).

### Cue-locked long-range α-synchrony

**Figure 3.**
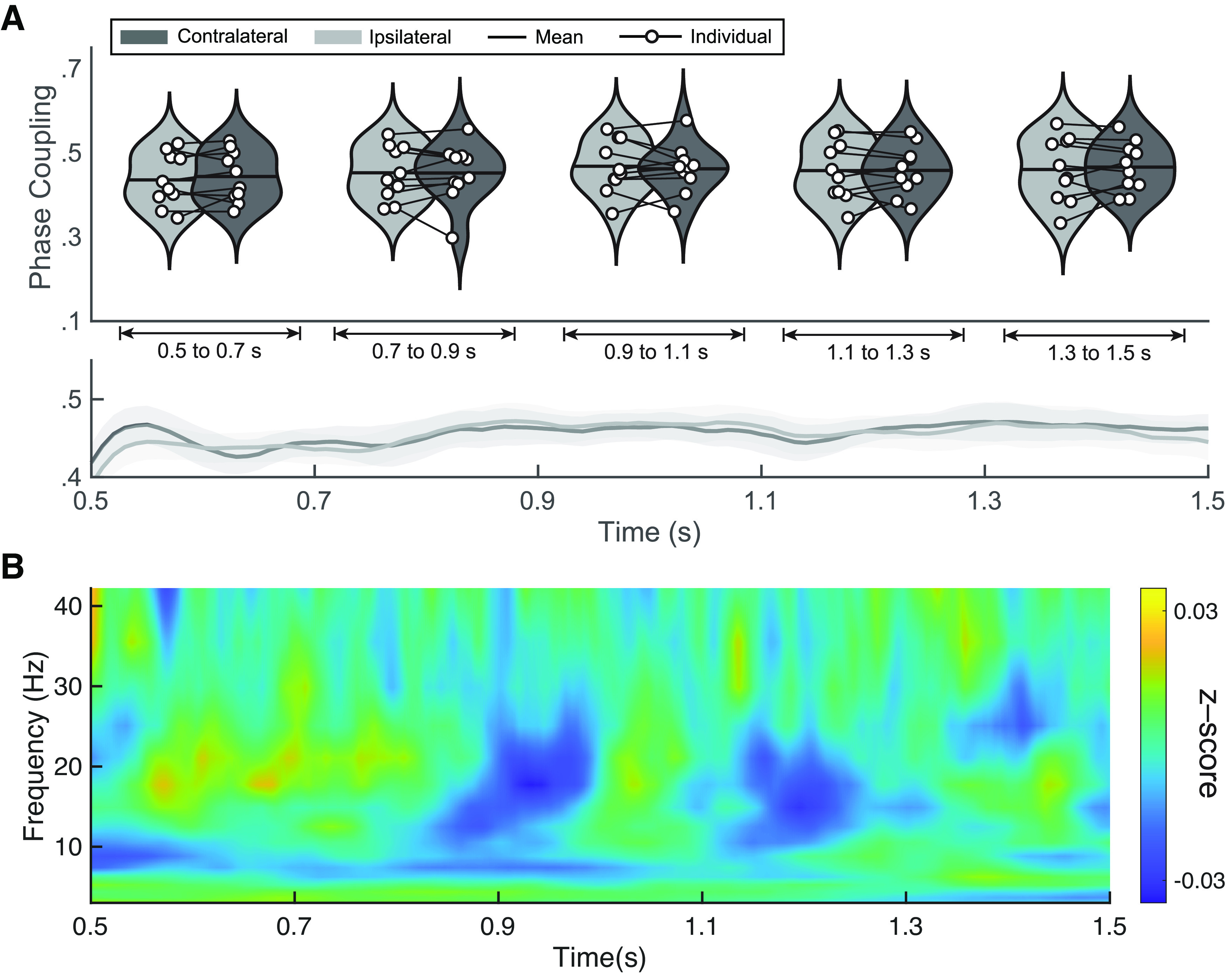
Cue-locked results. ***A***, Group-level results of upper-α phase locking value (PLV). Upper panel shows phase coupling for ipsilateral (light gray) and contralateral (dark gray) sides in time-windows of 200 ms from the cue-locked interval (500–1500 ms after cue presentation). Lower panel shows mean and standard error of the mean of the PLV values. Individual results are shown in Extended Data Figure 3-1. ***B***, Exploratory analysis of PLV differences. Group-level temporal evolution of the z-scored difference between contralateral and ipsilateral PLV for each frequency band (2.4–42 Hz with 16 logarithmic steps). Z-score values range from −0.03 to 0.03. Individual results are shown in Extended Data Figure 3-2.

In the previous section, we replicated the results as previously shown (Sauseng et al., 2005). The findings from here onwards correspond to original results to ascertain whether attention-based long-range connectivity during the attention-orienting period could be a reliable signal for BCI control. We explored the cue-to-target interval before target presentation (500–1500 ms after cue onset). Considering that the cue indicates the hemifield to which participants should voluntarily lateralize attention, differences in contralateral and ipsilateral connectivity may potentially emerge in this time window. So far, we have seen that attention shifts had significant consequences on behavior and target processing (post-target connectivity). At the group level, however, no significant difference between contralateral and ipsilateral connectivity in the upper α-band was found in any of the five 200-ms time windows considered in the cue-to-target period (see Fig. 3*A*). At the individual level, seven participants had a significant contralateral PLV increase in at least in one window (see Extended Data Fig. 3-1). However, only one participant (P10) showed this effect in all time windows and, furthermore, did not present a significantly higher contralateral connectivity in pretarget and post-target time windows of the target-locked analysis.

10.1523/ENEURO.0203-22.2023.f3-1 Extended Data Figure 3-1 Individual results of upper-α cue-locked PLV analysis. Violin plots represent the phase locking values (PLV) averaged over the five time windows (500–700, 700–900, 1100–1300, and 1300–1500 ms; *t* = 0 as cue appearance). Ipsilateral or contralateral scenarios are exhibited as either light grey or dark grey, respectively. *p <* *0.05, **p <* *0.01. Download Figure 3-1, EPS file.

10.1523/ENEURO.0203-22.2023.f3-2 Extended Data Figure 3-2 Individual results of cue-locked exploratory PLV analysis. Differences of contralateral to ipsilateral PLV are represented over frequencies (2.4–42 Hz in 16 logarithmic steps) as a percentage of change regarding the cross-frequency mean of each individual. Download Figure 3-2, EPS file.

We chose the upper α-band a priori given Sauseng et al. (2005)’s findings, as well as the effects in the target-locked analyses from the present dataset. However, we conducted additional analyses to explore other frequencies (between 2.4 and 42 Hz) in search of differences between contralateral and ipsilateral PLV (see Fig. 3*B*). Values were collapsed as the difference between both measures (contra-ipsi) and z-scored. Over time, neither clear trends across frequencies nor apparent increases were observed in contralateral or ipsilateral connectivity. Individual results showed the same trend and did not present relevant PLV patterns in any participant beyond those from upper α-band findings in P10 (see Extended Data Fig. 3-2).

### Classification

The results are hardly promising in generalizing the use of long-range connectivity for BCI control. However, BCI protocols are often very sensitive to individual patterns. Here, we intended to seek a proof-of-concept, from at least a single participant. With this goal in mind, we attempted single-trial classification, as either attended right or attended left, according to cue-locked connectivity patterns. We selected the participant (P10) for whom we found significant connectivity differences in the cue-to-target time window of the cue-locked analysis. The total number of trials was 338.

**Figure 4.**
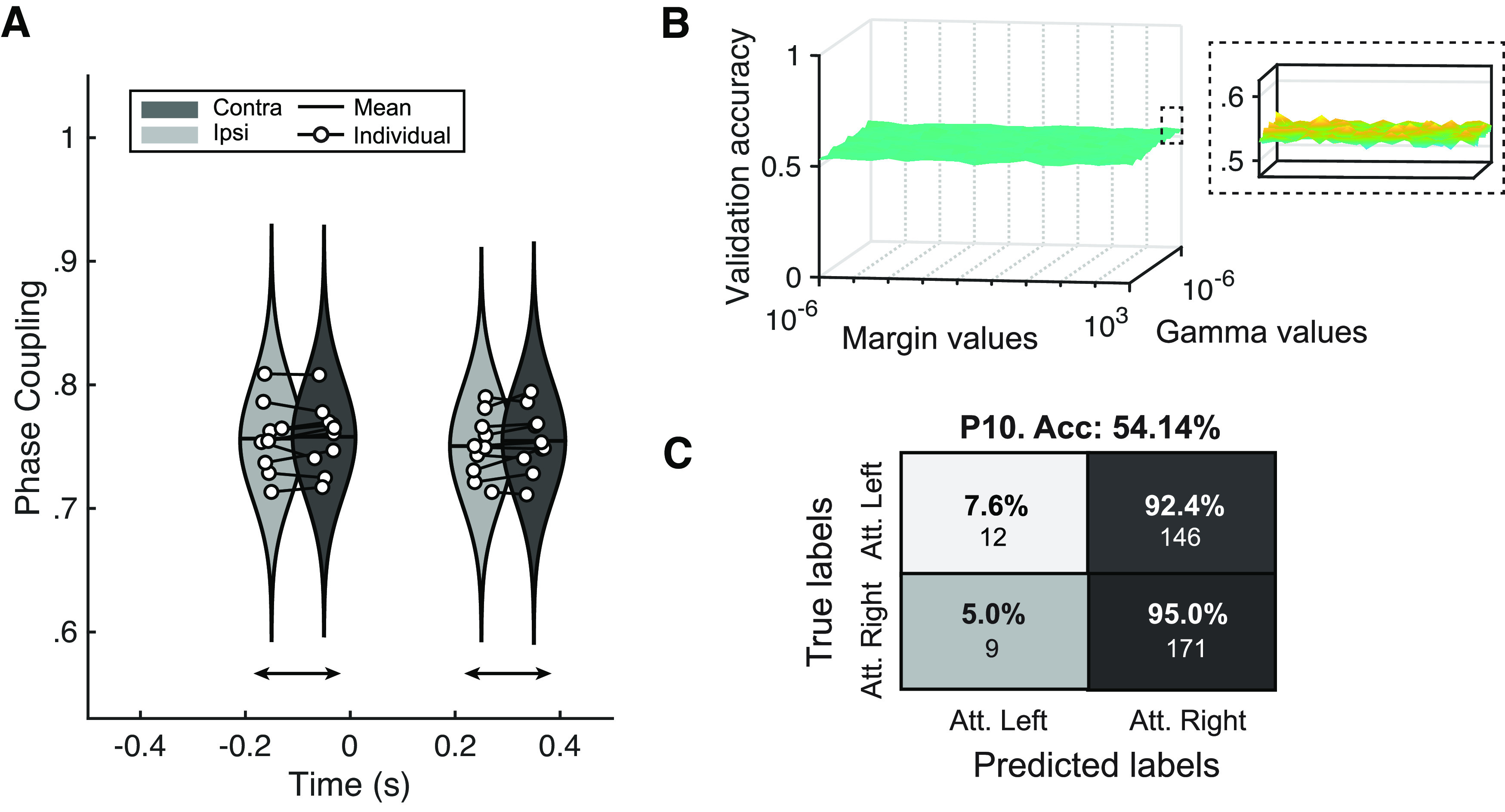
Classification outcomes. ***A***, Cross-time phase locking value (PLV) reality check. Replication of results from Figure 2 calculating PLV across time points rather than across trials. Individual results are shown in Extended Data Figure 4-1. ***B***, Optimization results of γ and margin parameters of the Gaussian kernel support vector machine. Ten-fold validation accuracies with varying margin values (*x*-axis) and γ values (*y*-axis). Inset shows a detailed view of the *z*-axis. ***C***, Confusion matrix of the classification outcomes for one participant. *y*-axis represents ground truth labels (attended right or attended left) and *x*-axis represents the classifier’s outcomes. Percentages represent the fraction of correctly classified trials of each condition (i.e., each row sums to 100%). Under the percentage is the gross number of classified trials. Results with additional classifiers such as shrinkage linear discriminant analysis (sLDA) and Riemannian minimum distance to the mean (RDMD) are shown in Extended Data Figure 4-2.

We conducted a validation of cross-time PLV in the target-locked window to understand whether this metric could replicate group-level differences between contralateral and ipsilateral networks found through cross-trial PLV. These results can be seen in Figure 4*A*. Statistical analysis showed no significant differences between contralateral and ipsilateral scenarios in either time window. Individual values were also nonsignificant (see Extended Data Fig. 4-1). Considering the large parametric landscape of SVM implementations, we optimized the γ and margin parameters of a Gaussian kernel (see Fig. 4*B*). From a qualitative perspective, no clear maximum validation accuracy values emerge from the landscape, although quantitative analysis identified minimum values of margin and γ to be used on the test set in every fold. The lack of a clear minimum suggests that the model may be unable to classify individual trials regardless of the parametric values.

10.1523/ENEURO.0203-22.2023.f4-1 Extended Data Figure 4-1 Individual results of target-locked cross-time PLV. Violin plots represent the phase locking values (PLV) obtained by calculating PLV as consistency throughout the pretarget (−200–0 ms) and post-target (200–400 ms) time windows. Ipsilateral or contralateral scenarios are exhibited as either light grey or dark grey, respectively. Download Figure 4-1, EPS file.

10.1523/ENEURO.0203-22.2023.f4-2 Extended Data Figure 4-2 Additional classifier analysis. ***A***, Shrinkage linear discriminant analysis. The leftmost panel reveals how classification error is not modulated by gamma parameter of number of predictors. The rightmost panel presents the confusion matrix. ***B***, Riemannian minimum distance to the mean classification results. Download Figure 4-2, EPS file.

Ten-fold cross-validation was conducted to maximize the available data and improve the classification accuracy. Single trials predicted as either attended right or attended left were contrasted with the actual cue direction in each trial. Classification outcomes are shown in Figure 4*C*, which resulted in virtually chance-level sorting (0.541). The confusion matrix displays the distribution of each class, revealing the skewed distribution of values toward attended right labels, which is far from the ideal clustering along the diagonal of the matrix. Finally, we employed two additional algorithms to classify both attended right and attended left trials. These consisted of shrinkage linear discriminant analysis (sLDA) and Riemannian minimum distance to the mean (RMDM), as they are shown to work well in small training sets (Lotte et al., 2018). Both decoding techniques yielded chance-level results (see Extended Data Fig. 4-2).

### Interhemispheric power imbalance

**Figure 5.**
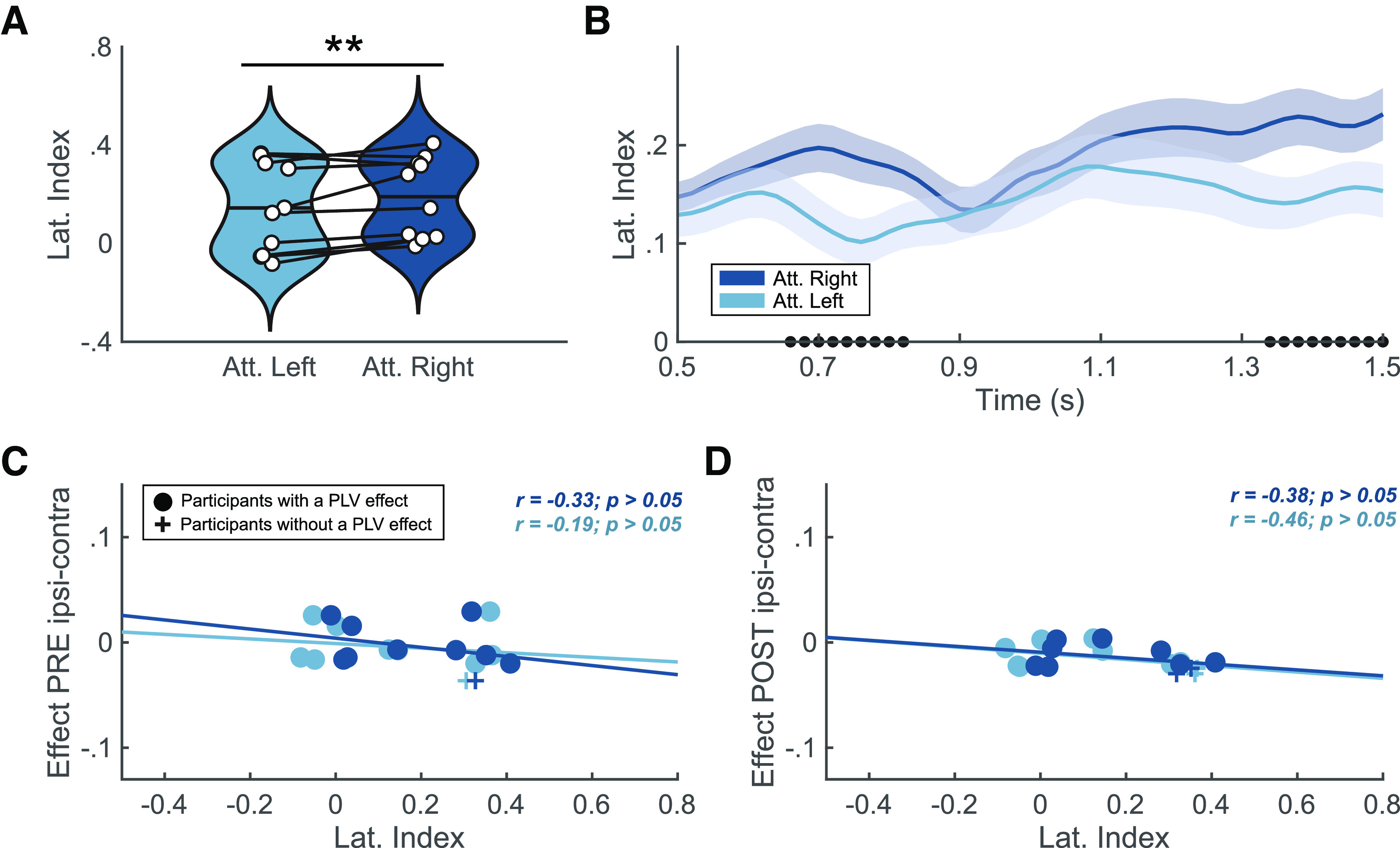
Lateralization index reality check. ***A***, Averaged lateralization index for attended left (light blue) and attended right (dark blue; *p < 0.05, **p < 0.01). White dots denote individual scores, and horizontal line indicates the group mean. ***B***, Lateralization index (mean ± standard error of the mean; SEM) over time. Solid lines and shaded areas represent mean and SEM interval, respectively. Dots on in the *x*-axis denote the significant difference over time between attended left (light blue) and attended right (dark blue) via cluster-based permutation test. Individual results are shown in Extended Data Figure 5-1. ***C***, ***D***, Lateralization indexes and the difference of contralateral to ipsilateral phase locking value (PLV) for attended left (light blue) and attended right (dark blue) at the pretarget window (***C***) and the post-target window (***D***). At the pretarget the correlations for attended right (*r* = −0.33, *p *>* *0.05) and attended left (*r* = −0.19, *p *>* *0.05) did not reach significance and neither did the correlations for attended right (*r* = −0.38, *p *>* *0.05) and attended left (*r* = −0.46, *p *>* *0.05) at the post-target window. Crosses denote participants with a significant effect in PLV contra-ipsi differences at the pretarget window (−200–0 ms; participant P05) and the post-target window (200–400 ms; P02 and P07). Dots represent the rest of the participants.

As a reality check on the dataset, we addressed whether there was a difference in the α-power interhemispheric imbalance between attended left and attended right trials. We performed the cue-locked analysis at the group level, using the Lateralization Index (LI) described by Thut et al. (2006; see Fig. 5*A*). On average, the lateralization index was significantly different between attended right and attended left in the expected direction (*p < *0.01, Cohen’s *d* = −0.8356). At the individual level, 7 out of the 10 participants showed a significant difference in lateralization index between the two attention conditions (*p < *0.05; see Extended Data Fig. 5-1). We also performed a time-resolved version of this analysis within the cue-to-target window. A cluster-based permutation test (Fig. 5*B*) showed significance within two time periods, from 0.66 to 0.82 s and 1.34 to 1.5 s. At the individual level, only for one participant (P01), the cluster-based permutation test revealed a significant cluster over time from 0.6 to 1 s (see Extended Data Fig. 5-1). These results are consistent with the results of previous studies (Thut et al., 2006; Tonin et al., 2012), at least at the group level. It is more challenging to compare single-subject data with other studies, as they are usually neither reported nor statistically analyzed.

10.1523/ENEURO.0203-22.2023.f5-1 Extended Data Figure 5-1 Individual results of lateralization index. Violin plots represent the averaged lateralized index for attended left (light blue) and attended right trials (dark blue) over the cue-locked time window. Shaded plots represent lateralization over time (mean ± SEM). Dots on in the x-axis denote the significant differences over time between attended left and attended right via cluster-based permutation test. *p <* *0.05, **p <* *0.01, ***p <* *0.001. Download Figure 5-1, EPS file.

Finally, we explored the potential correlation between α-power interhemispheric imbalance measured with the lateralization index and α-phase coupling for each attended location (see Fig. 5*C*,*D*). In the pretarget window (Fig. 5*C*), the correlations for attended right (*r* = −0.25, *p *>* *0.05) and attended left (*r* = −0.13, *p *>* *0.05) did not reach significance. Neither did the correlations for attended right (*r* = −0.44, *p *>* *0.05) and attended left (*r* = −0.42, *p *>* *0.05) at the post-target (Fig. 5*D*) window. Visual inspection indicated that participants showing an effect in PLV contra-ipsi differences are below the correlation fit in pretarget and post-target windows, suggesting that those participants have a more negative effect in PLV contra-ipsi differences.

## Discussion

The present study addressed the relationship between shifts in visuospatial attention and the lateralization of α-band coherence between frontal and parietal electrodes, to assess its feasibility as a control signal in BCI. Previous studies, using group-averaged multi-trial analyses, found increased long-range α-synchronization in the hemisphere contralateral to the attended hemifield, and suggested that it reflects top-down mechanisms of visual spatial attention (Sauseng et al., 2005; Doesburg et al., 2009). We reasoned that if contralateral to ipsilateral differences in synchronization would emerge as a result of endogenous top-down mechanisms, they should be present following cue presentation as participants shift their attention. This hypothesis stems from how instructing participants to shift their attention laterally before target appearance engages frontoparietal visual processing pathways (Hopfinger et al., 2000; Corbetta and Shulman, 2002; Asplund et al., 2010). Here, we sought proof that long-range neural synchronization engaged in this network could be used for BCI control on a trial-by-trial basis.

In attention-orienting protocols, the cue-to-target period offers the possibility of implementing a BCI control in anticipation of the target appearance. This would open the possibility of designing active BCI systems controlled by the user’s voluntary decision to attend left/rightward covertly. Therefore, our study employed long-range α-synchronization in the frontoparietal network (FPN) as means to investigate whether this brain measure could potentially discriminate attended locations of the left/right visual field.

We found significant group-level differences in contralateral to ipsilateral long-range α-synchronization around target onset, replicating Sauseng et al. (2005). These results demonstrate the involvement of lateralized long-range α-synchrony along the FPN during the post-target period and especially reveal the potential of EEG to grasp these effects, at the group level. However, similar differences in frontoparietal synchrony were not observed during the cue-to-target time window, which was the time of interest for BCI purposes. We also extended the cue-locked analysis to other frequencies outside the α-band, with equally negative results. Finally, given the high individual variability of single-trial analysis outcomes, we attempted to classify the individual trials of one selected participant for whom significant synchronization differences following cue presentation were found, as a benchmarking process. The results nevertheless rendered chance-level classification. Below, we discuss how these results may be influenced by various methodological aspects (e.g., different time windows, classifier’s input metric) and how they fit into state-of-the-art literature. Please note that because the focus of our study was on single-trial analysis, the sample size was relatively small for the group analyses (*n* = 10). Although this sample size was sufficient to confirm previous findings on long-range α-synchronization and lateralization index (Sauseng et al., 2005; Thut et al., 2006), the negative results of the group analyses should be interpreted with caution.

### Frontoparietal network synchronization characterizes visuospatial attention

A result from our study is that long-range α-synchronization within the FPN was associated with the consequences of visuospatial attention orienting, in line with its putative role in this cognitive process (Siegel et al., 2008; Doesburg et al., 2009; Jensen et al., 2015; Sacchet et al., 2015). We observed significant increase in contralateral versus ipsilateral upper α coherence for targets appearing at the attended location. According to current attention theories, the mechanism underlying this finding may be inherently related to top-down processing. More specifically, frontal regions such as the frontal eye fields (FEF) and the intraparietal sulcus (IPS) may modulate attention by causing a state of α-band desynchronization in the visual cortex contralateral to attended hemifield (Kastner and Ungerleider, 2000; Corbetta and Shulman, 2002; Capotosto et al., 2009; Marshall et al., 2015; Helfrich et al., 2018). This explanation further aligns with the well-established evidence that contralateral α-power suppression (also reproduced in our results) enables visual stimuli processing in the attended location (Yamagishi et al., 2003; Babiloni et al., 2006; Thut et al., 2006; Klimesch et al., 2007; Doesburg et al., 2009; Foxe and Snyder, 2011; Lange et al., 2013), and that cyclic phase-dependent inhibition in low-level visual cortex dictates behavioral performance (i.e., reaction times; Haegens et al., 2011; Klimesch, 2012; Jensen et al., 2014; Samaha et al., 2015; VanRullen, 2016). Both accounts fit with the idea that local α-power and long-range α-synchronization may have separate roles in attention and perception (Palva and Palva, 2007, 2011; Sadaghiani and Kleinschmidt, 2016; Bonnefond et al., 2017).

Our results of the increased contralateral synchronization within the FPN replicate the work of Sauseng et al. (2005) and validate our methodology and analysis pipeline (e.g., time-frequency analysis, synchronization metric), setting the ground for the intended proof of concept test regarding transference to BCI. However, lateralized frontoparietal connectivity patterns in attentional and perceptual disposition remain challenged in the literature together with the role of α power/phase (Ruzzoli, Torralba et al., 2019; van Diepen et al., 2019; Antonov et al., 2020; Keitel et al., 2022). Lobier et al. (2018) found that α-synchronization was associated with visuospatial attention but revealed distinct lateralization patterns regarding the visual system and top-down attentional networks. They showed stronger ipsilateral synchronization within the visual system (in line with Siegel et al., 2008; Doesburg et al., 2009) but no consistent lateralization in long-range networks, suggesting their different involvement in visuospatial attention. A study by D’Andrea et al. (2019) found a modulation of frontoparietal α- β cross-frequency synchronization during attention orienting, but not in α-synchronization alone. Further, this cross-frequency connectivity pattern was strongly associated with right hemisphere frontal dominance, in line with Heilman and van den Abell (1980) and Zago et al. (2017). This finding agrees with previous evidence of the crucial role of the right FEF in top-down attentional modulation (Silvanto et al., 2006; Hung et al., 2011; Esterman et al., 2015; Veniero et al., 2021), supported by evidence using TMS (Capotosto et al., 2009). In light of this evidence and our results, the exact relationship between contralateral frontoparietal α-synchronization and shifts in attention orienting is still unclear. Positive findings, however, such as the ones in the present study using a target-locked analysis, represent a basis for exploring earlier time windows capable of shedding light on the mechanism underlying FPN α-synchronization.

Correlations between long-range α-synchronization and individual reaction times in visuospatial tasks suggest this neural correlate may be observable at a single-subject level (Lobier et al., 2018). However, significant group-level target-locked dynamics of increased synchrony did not transfer to all individuals in our study. The observed variability may be partially explained by individual anatomic differences in the neural substrate of attention (e.g., superior longitudinal fasciculus; Marshall et al., 2015). Findings employing magnetic resonance imaging (MRI) suggest that volumetric differences in these structures impact local visual cortex oscillations, leading to variability in EEG traces (Marshall et al., 2015; D’Andrea et al., 2019). However, this variability of individual results is challenging to set in the perspective of previous research simply because published studies do not report single-subject statistics. Ultimately, the outcomes of this study leave an incomplete understanding of whether there is a reliable group effect that does not extend to all individuals or, contrarily, whether individual effects of specific participants are large enough to induce a group-level finding in previous research.

### Lateralized patterns of α-synchronization appear in target-locked but not cue-locked analysis

In our study, long-range α-synchronization presented contralateral increases at the post-target (200–400 ms, with *t* = 0 as target appearance) and the pretarget window (−200–0 ms), but only the former time window resulted significantly. This result is slightly different from Sauseng et al. (2005), who observed significant increases in contralateral synchronization within the FPN network at both time windows. However, the numerical differences were in the same direction in both studies, leaving the possibility that statistical significance be just because of a lack of statistical power. Another potential explanation for the absence of significant findings at the pretarget window may be the difference in experimental paradigms. The task employed here had a longer postcue interval ranging from 2000 to 2500 ms (jittered between trials), compared with Sauseng et al. (2005; i.e., 600–800 ms). If participants shifted attention at varying times from cue onset up to target appearance, this might explain why we could not capture the effect in anticipatory visuospatial attention.

In cueing paradigms, bottom-up integration of cue information through sensory pathways precedes top-down modulation of visuospatial attention (Simpson et al., 2011). The time course of voluntary directed attention is thought to begin only after 150 ms from cue onset and involves frontal regions approximately after 350 ms. Furthermore, from 400–500 ms onwards, frontal and parietal regions are thought to be involved in attentional shifting and target discrimination (Simpson et al., 2011). Thus, if the FPN does present direction-specific synchronization, we anticipated this would appear from ∼500 ms after cue onset onwards. Contrary to what we expected, we did not observe any significant contralateral to ipsilateral differences in the cue-to-target time windows (500–1500 ms after cue onset). Previous studies employing a similar time window showed lateralization patterns in parietal regions in α and β bands (Siegel et al., 2008; Pantazis et al., 2009) and frontoparietal lateralization in low and high-frequency bands (Green and McDonald, 2008; Gregoriou et al., 2009). Therefore, we extended our cue-locked analysis to other frequencies but again obtained no significant contralateral to ipsilateral differences. Note that PLV values were averaged across 200-ms windows, and this excludes, to a certain extent, the confound of frontal and parietal regions having different activation over time. Altogether, despite the evidence across multiple frequencies of synchronization in the cue-to-target time window, we did not find patterns of lateralized cue-locked connectivity within or outside the α-band.

Our negative results in the cue-locked analysis may align with the notion that late periods after cue onset are associated with direction-specific activity in parieto-occipital regions but not in frontal regions (e.g., FEF; Doesburg et al., 2009; Simpson et al., 2011). Long-range α-synchronization may, therefore, be associated to an initial shift of attention (shortly after cue presentation) and later (close to target presentation) to attention maintenance at the directed hemifield (Hopfinger et al., 2000; Kastner and Ungerleider, 2000; Grent-'t-Jong and Woldorff, 2007; Lobier et al., 2018). This idea resonates with the essential question formerly posed by Sauseng et al. (2005), debating whether frontal involvement in long-range α-synchronization is a causative or consequential correlate of posterior activation. Furthermore, it motivated the exploration of cue-locked intervals where bottom-up and top-down processing may have elicited stronger effects on α-band synchronization.

Finally, to ensure participants correctly lateralized their attention during the cue-to-target interval, we conducted a reality check by calculating the α-power imbalance using the lateralization index over this period (Thut et al., 2006). There was a clear difference in the averaged lateralization index between 500 and 1500 ms at group level. We further employed the lateralization index to perform an exploratory analysis of its relationship with the difference in α-synchronization between contralateral and ipsilateral networks. Considering lateralized local α activity and lateralized long-range α-synchronization are both relevant in successful attention orienting, we explored whether these two mechanisms would have had a significant positive correlation. Therefore, individuals with high lateralization index should also present lateralized synchronization within the FPN. In contrast to our expectations, there was no significant correlation between these two metrics, neither at the pretarget nor the post-target time windows.

Ultimately, we did not observe a significant increase in contralateral long-range α-synchronization in the five 200-ms bins following cue onset. This time frame offered potential as it occurs much before target appearance and could be robustly employed in a covert visuospatial BCI decoder. By expanding our analysis to several frequencies and carrying out the aforementioned reality checks, we conclude that PLV measured from EEG may not serve as a reliable metric in capturing direction-specific synchronization from frontal to posterior regions, despite this evidence being present in parietal to occipital synchrony (Doesburg et al., 2009).

### EEG estimates of long-range α-synchronization may not serve as a reliable control signal for BCI

The use of long-range α-synchronization to decode attentional direction yielded chance-level results. We employed 200-ms time bins of contralateral and ipsilateral FPN connectivity as input in an SVM classifier. Nonlinear SVMs are widely employed in decoding cognitive neural correlates of behavioral states (Lotte et al., 2007). Furthermore, SVMs outperform other classifiers, such as artificial neural networks, nonlinear Bayesian estimators, and recurrent reservoir networks (Astrand et al., 2014a). We also employed sLDA and RMDM classifiers, as they have low computational cost, require small training sets, and perform well in real-time applications (Lotte et al., 2018), with no success.

Prior work using SVMs, mainly centered around primate models and invasive recordings, successfully decoded the attentional spotlight from frontal sites (Esghaei and Daliri, 2014; Tremblay et al., 2015; Gaillard et al., 2020). Clearly, these methods (i.e., LFP, intracranial-EEG) have a higher signal-to-noise ratio (SNR) compared with noninvasive imaging. However, the objective of the present study was to offer a BCI proof of concept using α-synchronization as a control signal. Therefore, a noninvasive and portable technique must be employed. Other noninvasive modalities such as functional magnetic resonance imaging (fMRI), where the temporal resolution is too low for real-time implementations, or magnetoencephalography (MEG), where the equipment is expensive and requires a magnetically shielded room (as fMRI), have limited potential transfer in out-of-lab applications. Contrarily, EEG is an affordable imaging modality with a straightforward setup which provides high temporal resolution and portability. However, the inconvenience of using EEG is a low spatial resolution and a low SNR. Despite this, decoders have been commonly employed in EEG-BCI design employing parieto-occipital power changes in α-band activity to predict covert visuospatial attention tasks (van Gerven and Jensen, 2009; Treder et al., 2011; Tonin et al., 2013). The integrated approach between frontal and parieto-occipital attentional decoding based on α-synchronization, however, has not been attempted. Here, we found that cue-locked synchronization enclosed in the FPN α-band is insufficient to determine the attentional location at EEG single trial level. This may be because of an inherent lack of connectivity in the cue-to-target interval, or else more likely, the poor sensitivity of the EEG to register synchronization patterns.

Another potential reason to explain the failed classification of cue-locked FPN connectivity at single-trial level may be the change in PLV calculation from trial-average to single-trial. Standard cognitive research employs multiple trials to estimate consistent findings on electrophysiological markers (M/EEG). Instead, BCIs need robust and accurate estimates in a single-trial fashion and thus require a trade-off between spatial (i.e., single-channel decoding is preferred) and temporal resolution. PLV is a measure of consistency across multiple trials and cannot serve as a single-trial control signal. Therefore, we computed PLV across time points within the same trial. This new measure is also referred to in the literature as the intersite phase clustering (ISPC) and may represent a different underlying process than that captured by classic PLV (Cohen, 2015). This prompts the question of whether long-range α-synchronization is incapable of decoding the attended location, or rather the single-trial nature of IPSC over time is responsible for this.

In sum, long-range α-synchronization within the FPN estimated with EEG may not serve as a control signal for BCI. This limitation may be because of incomplete information on neural correlates because of the lack of cross-frequency analysis or the computational techniques surrounding ISPC over time.

In conclusion, we found direction-specific contralateral patterns of upper α-synchronization (i.e., PLV) within the FPN following target appearance in a covert visuospatial task. This finding, however, did not extend to pretarget or cue-to-target time windows. The modulatory role of α-synchronization in anticipatory attention through frontal, parietal and occipital regions suggests that PLV may not constitute a reliable metric for this top-down visual processing. Furthermore, chance-level classification resulting from using this metric in an SVM indicates that long-range α-synchronization computed with EEG may not be a suitable control signal for BCI.

## Synthesis

Reviewing Editor: Niko Busch, Westfalische Wilhelms-Universitat Munster Decisions are customarily a result of the Reviewing Editor and the peer reviewers coming together and discussing their recommendations until a consensus is reached. When revisions are invited, a fact-based synthesis statement explaining their decision and outlining what is needed to prepare a revision will be listed below. The following reviewer(s) agreed to reveal their identity: Domenica Veniero, Marie-Constance Corsi.

# Review synthesis

Both reviews highlight the merit of this study, but they also agree on the need to improve the manuscript. Specifically, both reviewers suggest adding additional information about the signal processing methods. Moreover, I generally agree with the reviewers’ concerns that it would help the manuscript to demonstrate that the signal processing methods were indeed sensitive enough in order to substantiate the negative finding of your study. This would involve complementing your methods with additional analyses, as suggested by reviewer 2 (especially the state-of-the-art methods described in the Lotte et al, 2018 paper). Finally, I would like to ask you to consider making data and code publicly available.

# Reviewer 1

In this feasibility study, the authors looked for a signature of attentional shift direction that could be later used for BCI. Specifically, they test the idea that long-range connectivity in the alpha-band between frontal and parietal electrodes might show a lateralization similar to the well-known alpha power lateralization over occipito-parietal areas. To this aim, they analyzed data collected during a Posner task with an auditory cue and visual target. They found a lateralization of alpha connectivity after the target presentation, but no effect on cue-to-target interval. Decoding analysis confirmed this finding. The authors conclude that at least on a single-trial basis, the EEG connectivity fails to distinguish between attended and unattended side.

This is an interesting and well-designed work that I enjoyed reading. Experimental design and analysis are sound and clearly described. I only have a few points (mainly minor) I would like the authors to address to shed light on some sections of the paper.

Below are my comments (in no particular order).

1- In the intro, there is a brief section on communication through coherence (fries, 2005) and I was just wondering if the authors could cite some papers in line 78, where they say there is overwhelming evidence in support of this idea.

2- Were catch trials included in the task? Linked to this point, I was also wondering if the participants’ performance could be a potential issue and difference from Sauseng et al 2005. In the present paper, the performance on the unattended side is at chance level. If participants cannot perceive the stimulus when it is presented at the unattended location, there might be no need to inhibit the information coming from that hemifield (after some trials) and therefore the ipsi-vs contralateral difference becomes irrelevant in the cue to target interval (figure 2b).

3- How were the electrodes included in the analysis chosen? Also, I would suggest replacing ROI with EOI (electrodes of interest).

4- Related to the choice of ROIs, I think this could be a limitation of the study. The frontal area is represented by just 3 electrodes that cover both hemispheres. However, the electrodes to brain structure correspondence are extremely variable across participants and more so for areas such as FEF. Also, there are some studies that seem to indicate that the right and left frontal areas might be differently connected (anatomically and functionally) to parieto-occipital areas. Why limit the analysis to 3 electrodes?

5- It is not always clear which analysis is replicating Sauseng et al 2005.

6- Why were different cycles used for the 2 different Morlet (lines 251 vs 190)?

7- In the discussion section (line 441), the authors summarise their results and say they found “significant group-level differences in contra- to ipsilateral long-range alpha-synchronisation around target onset time”. They conclude this is evidence of the involvement of long-range ⍺-synchrony during attentional orienting. However, the results only indicate a significant increase in coherence after the target has been presented. Why would this still be about attentional orienting?

8- I could not open figure 5.1

9- Could the authors explain what they mean by spatially distributed changes in coherence?

10- I am not sure I could follow the section that supports the role of the FEF in attentional shift that is part of the discussion.

# Reviewer 2

(1) General comments to the authors

The study deals with an interesting question associated with the search for alternative features in the context of BCI, and more particularly, for systems based on neural correlates of covert visuospatial attention (CVSA). The main hypothesis was that features based on long-range alpha-synchronization will be suitable for BCI. For that purpose, the authors replicated the analysis pipeline of Sauseng and colleagues on a cohort of 10 participants. The authors could elicit lateralized pattern of alpha phase coupling between frontal and parieto-occipital regions. Unfortunately, they could not achieve a high classification accuracy with these features.

The authors addressed a crucial question for the BCI domain, that is the use of features that could better capture brain functioning and, that could track its interconnected nature. Even though providing negative results is of interest to make progress in science, the lack of comparisons with classical methods and the lack of thorough analysis of the dataset make the authors’ main statement less insightful.

(2) Specific Comments (major)

Please find bellow some questions, comments, and suggestions which may improve the paper:

It must be acknowledged that the authors make the effort to consider the subjects’ specificity via the IAF computation that is particularly crucial when tracking modulations of the brain activity in the alpha band.

Some information is missing for the EEG preprocessing:

- On average, how many independent components were removed? What was the judgement criterion to select them? Was it automatic?

- Why discarding trials with EOG components after performing an ICA?

- On average, how many channels were removed? Were they located above specific regions?

- Why applying several filtering steps (that could potentially distort the signal) if the authors were interested in the alpha band?

- I found quite misleading to write that this is a replication of a previous study and then, applying different time windows, knowing that this information is crucial to perform the classification.

- Using the PLV measure is totally of interest, but it lacks a presentation of the rationale its use, given that other metrics could also capture phase synchrony and potential non-linearities of the brain activity (e.g. those based on phase-lagged index PLI [1], Phase Linearity Measurement PLM [2])

- Knowing that some functional connectivity estimators are more sensitive towards volume conduction. Did the author consider working in the source space to reduce the effect of the volume conduction that could lead to spurious connections? Furthermore, it could enable a better interpretation of the results.

- What is the rationale behind the use of the SVM? I perfectly understand that it is used for classification in the BCI domain but other classifiers could be more suitable (shrinkage LDA, Riemannian classifiers, ... please [3] see for a complete overview of the literature).

- The most concerning point here is the lack of comparison with the state-of-the art methods (both from the features and from the classifiers perspectives). This is particularly crucial to state if one new framework provides an improvement of the BCI classification. This is also true to provide insights when one is obtaining negative results since it would enable to establish if it is due to the proposed methods and/or the quality of the dataset itself.

References

[1] C. J. Stam, G. Nolte, et A. Daffertshofer, « Phase lag index: assessment of functional connectivity from multi channel EEG and MEG with diminished bias from common sources », Hum. Brain Mapp., vol. 28, no 11, p. 1178‑1193, nov. 2007, doi: 10.1002/hbm.20346.

[2] F. Baselice, A. Sorriso, R. Rucco, et P. Sorrentino, « Phase Linearity Measurement: A Novel Index for Brain Functional Connectivity », IEEE Trans. Med. Imaging, vol. 38, no 4, p. 873‑882, avr. 2019, doi: 10.1109/TMI.2018.2873423.

[3] F. Lotte et al., « A Review of Classification Algorithms for EEG-based Brain-Computer Interfaces: A 10-year Update », J. Neural Eng., févr. 2018, doi: 10.1088/1741-2552/aab2f2.
